# Unraveling the formation mechanism of graphitic nitrogen-doping in thermally treated graphene with ammonia

**DOI:** 10.1038/srep23495

**Published:** 2016-03-22

**Authors:** Xiao-Fei Li, Ke-Yan Lian, Lingling Liu, Yingchao Wu, Qi Qiu, Jun Jiang, Mingsen Deng, Yi Luo

**Affiliations:** 1 School of Optoelectronic Information, University of Electronic Science and Technology of China, Chengdu, Sichuan, 610054, China; 2 Division of Theoretical Chemistry and Biology, School of Biotechnology, Royal Institute of Technology, S-106 91 Stockholm, Sweden; 3 Hefei National Laboratory for Physical Sciences at the Microscale and Synergetic Innovation Center of Quantum Information and Quantum Physics, University of Science and Technology of China, Hefei, Anhui 230026, China; 4 Guizhou Synergetic Innovation Center of Scientific Big Data for Advanced Manufacturing Technology, Guizhou Education University, Guiyang, 550018, China

## Abstract

Nitrogen-doped graphene (N-graphene) has attractive properties that has been widely studied over the years. However, its possible formation process still remains unclear. Here, we propose a highly feasible formation mechanism of the graphitic-N doing in thermally treated graphene with ammonia by performing *ab initio* molecular dynamic simulations at experimental conditions. Results show that among the commonly native point defects in graphene, only the single vacancy 5–9 and divacancy 555–777 have the desirable electronic structures to trap N-containing groups and to mediate the subsequent dehydrogenation processes. The local structure of the defective graphene in combining with the thermodynamic and kinetic effect plays a crucial role in dominating the complex atomic rearrangement to form graphitic-N which heals the corresponding defect perfectly. The importance of the symmetry, the localized force field, the interaction of multiple trapped N-containing groups, as well as the catalytic effect of the temporarily formed bridge-N are emphasized, and the predicted doping configuration agrees well with the experimental observation. Hence, the revealed mechanism will be helpful for realizing the targeted synthesis of N-graphene with reduced defects and desired properties.

Chemical doping is a powerful technique to tailor the properties of carbon materials^1^,^2^,^3^,^4^. Recently the substitutional nitrogen-doping (N-doping) has attracted wide interests^2^,^5^,^6^,^7^ for its ability to tune and introduce new properties into graphene^8^ that considerably broadens the promising applications of graphene including supercapacitors^2^,^3^,^9^, solar fuel^6^,^10^, lithium-ion batteries^11^, molecular sensors^12^, electromagnetic devices^13^,^14^,^15^, and metal-free electrocatalysts for oxygen reduction reactions (ORR)^6^,^10^,^16^,^17^,^18^.

Nitrogen-doped graphene (N-graphene) can be fabricated by either direct-synthesis^4^,^5^,^6^,^17^,^19^,^20^ or post-synthesis^2^,^7^,^9^,^12^,^13^,^16^,^21^,^22^,^23^ methods. For direct-synthesis, N-doping is formed during the growth of graphene^4^,^5^,^6^,^19^ that can be explained by the established growth mechanism of graphene. For post-synthesis, N-doping is formed by post-treating the pre-prepared graphene^7^,^12^,^13^,^21^ or graphene oxide^2^,^9^,^16^,^22^,^23^ with N-precursors. Although several crucial factors such as the substrate, temperature, and pressure that seriously influence the N-doping process have been identified both in experiments and theories^6^,^8^,^13^, the synthesis of high-quality N-graphene with desired structures and properties is still not achieved. Measurements indicate that various N-species including pyrazole-N^22^, pyrrolic-N^2^,^16^,^21^, pyridinic-N^17^,^21^,^24^, and graphitic-N (quaternary-N)^5^,^10^,^12^,^13^,^17^,^25^ are formed, as presented schematically in Fig. 1. These N-species have essentially different effects on the carrier concentration and result in distinct electronic structures of the N-graphene^5^,^6^,^8^,^21^,^24^. Thus, to improve the performance of the N-graphene in various applications, a high content of desired N-species is always expected.

For the N-graphene obtained in some experiments^5^,^6^,^7^,^12^,^13^,^17^,^19^,^21^, most of N-dopants present in two major N-species of pyridinic-N and graphitic-N. The former forms normally at nanoholes or the edge of graphene^6^,^24^. A high content of pyridinic-N means the existence of a large amount of nanoholes that will largely reduce the conductivity of graphene^6^,^26^. Due to the existence of dangling bond, bare pyridinic-N is very reactive and may exist stably only in high vacuum^27^,^28^. Thus, although pyridinic-N has a novel electronic structure, it is not an effective promoter for many practical applications as reported^6^,^24^,^29^. The latter holds a higher thermal stability due to incorporated into graphene network. It is reported that graphitic-N plays paramount importance in various promising applications such as ORR^17^,^28^,^30^, molecular sensors^12^, water oxidations^10^,^31^, and electromagnetic devices^13^,^15^. For instance, Li *et al.* have reported that a high ratio of graphitic-N/pyridinic-N can provide a promising activity for ORR^17^. Thus, to achieve a high content of graphitic-N in N-graphene is expected. A detailed understanding of the formation mechanism of graphitic-N is highly desired.

Post-treatment of graphene with N-precursors has been proved a practical means to achieve high-quality N-graphene which is enriched in graphitic-N but small amounts of defects^7^,^12^,^13^. For instance, Lv *et al.*^12^ have reported that in their N-graphene obtained via thermal treatment of graphene with ammonia (NH_3_), most of the N-dopants (80%) are graphitic-Ns. It is intuitively clear that graphitic-N forms by merging N atoms into the defect region of graphene. First principles calculations have also proved that the occurrence of N-doping at defects of graphene is energetically favorable^27^,^32^,^33^, but the formation dynamics still need to be established.

In this work, by performing *ab initio* molecular dynamics (ab-MD) simulations at experimental conditions, we investigate the interaction of the widely used N-precursor of NH_3_ with the graphene of different structures. A highly feasible formation mechanism of graphitic-N is revealed, the defect-configuration relationship is established, the dynamics of the simultaneous formation of graphitic-N and the healing of the defect is visualized, and the important factors which dominate the formation process is emphasized. Thus, our simulations give new insights in N-graphene chemistry and will certainly help to achieve the targeted synthesis of N-graphene with reduced defects and desired doping structures.

## Results

**Figure 2:**
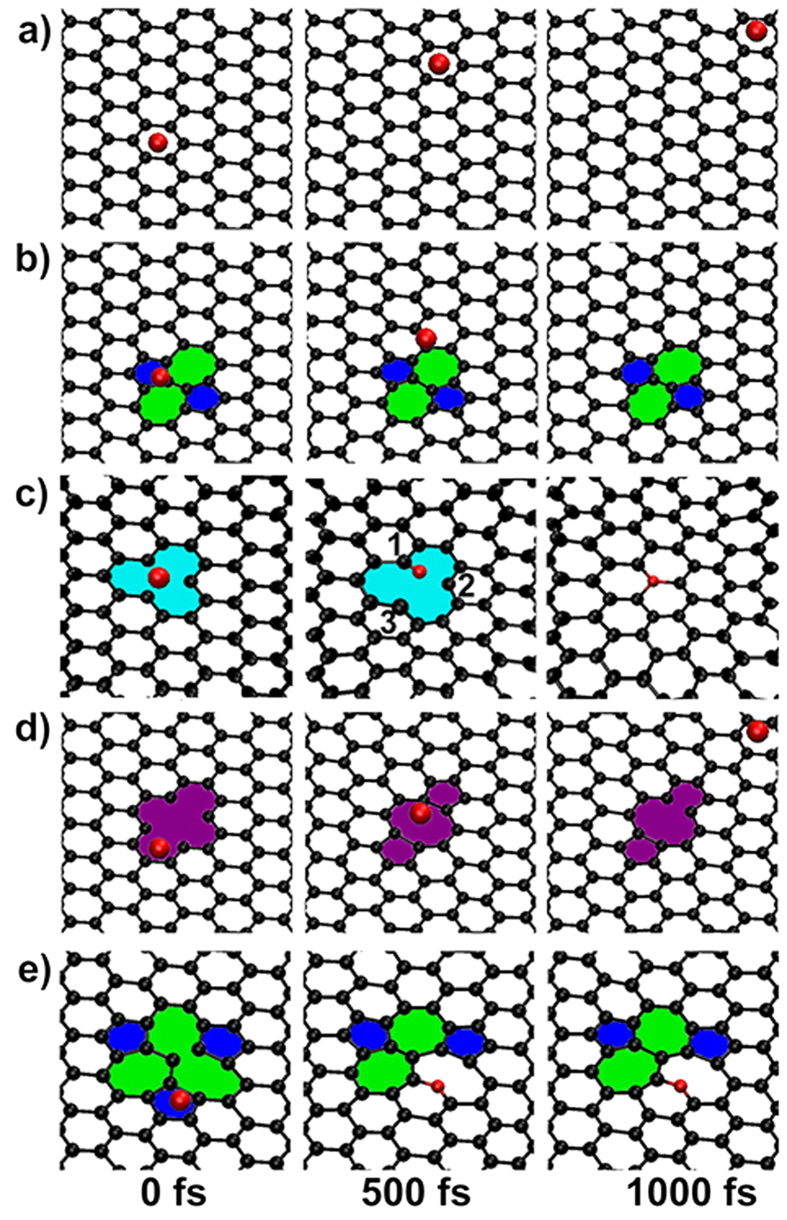
Interaction of a N atom with the graphene of different structures. The simulation snapshots for (**a**) perfect graphene, and imperfect graphene of (**b**) a native point defect of SW(55-77), (**c**) SV(5-9), (**d**) DV(5-8-5), and (**e**) DV(555-777), respectively. The display window is moved close to the active site of the used large supercell for clarity.

As a precondition to occur N-doping, N-sources should be trapped by graphene. We first examined the possibility to trap N atoms at the perfect region of graphene at experimental conditions. A temperature range from 700 to 1000 °C with a step of 50 °C has been used in the simulations, and the snapshots for 850 °C are plotted in Fig. 2(a). One can see that the N atom drifts freely above the surface and moves out of the sight at the end of simulation. The similar situation is observed at the simulations for other temperature. Thus, the probability of occurring N-doping at the perfect region of graphene at experimental conditions is very small due to the low activity of graphene.

Structural defects like Stone-Wales (SW), vacancies, nanoholes, grain boundaries, as well as the edges of graphene are known to possess a relative higher activity^26^,^27^,^34^. Considering that graphitic-N is merged into graphene, we examined the possibility of trapping N atoms by the commonly native point defects of SW(55-77), single vacancy SV(5-9), as well as divacancy DV(5-8-5) and divacancy DV(555-777), respectively. As illustrated in Fig. 2(b–e), the N atom is unlikely to be trapped by the SW(55-77) and DV(5-8-5), but it is trapped by the SV(5-9) and DV(555-777) under the same condition. The trajectories for SV(5-9) show that the N atom moves directly to the defect region and adsorbs on the top-site of the num-1 C atom. It then merges into the defect to form a graphitic-N which heals the defect perfectly. While for DV(555-777), the N atom first adsorbs on the bridge-site of the C-C bond shared by a 5–7 pair and then merges into the graphene layer to form a pyridinic-N only which can not heal the defect.

From the structural point of view, to heal a divacancy requires two adatoms. We have carried out simulations to study the interaction of two N atoms with a DV(5-8-5) and DV(555-777), respectively. Remarkably, only the DV(555-777) has trapped the two N atoms as expected (see Figure S2 in Supplementary Information). Trajectories show that one N atom merges into the defect region to form a pyridinic-N, but the other adsorbs and keeps at the bridge-site. Although the two N atoms are well trapped by the DV(555-777), the defect cannot be healed at all, even the simulation time has been extended to 3 ps.

In sharp contrast to the widespread intuition that a variety of defects in graphene can provide rich active sites for N-doping, our simulations demonstrate clearly that among the commonly native point defects of graphene, only the SV(5-9) and DV(555-777) have the ability to trap N atom, but it seems not always result in the formation of graphitic-Ns. Moreover, the decomposition of NH_3_ in thermal treatment experiments is complicated, which may involve various reactions and the products of -NH_2_, -NH-, and -N are possible. N atom is only the simplest model for the possible N-sources. We continued our simulations to investigate the interaction of a DV(555-777) and SV(5-9) with the N-containing groups of NH_3_, -NH_2_, and -NH-, respectively.

**Figure 3:**
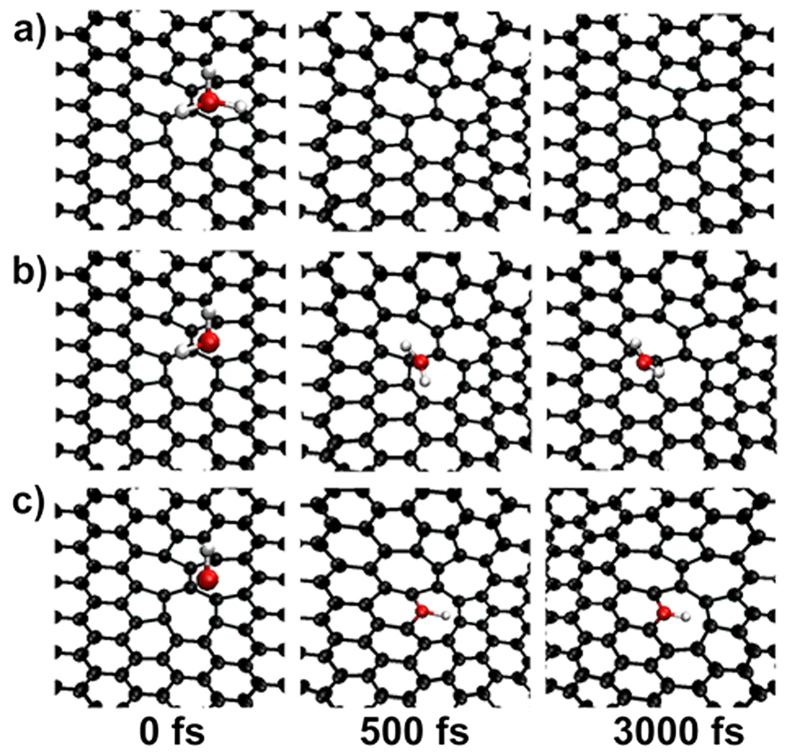
Interaction of a DV(555-777) with different N-containing groups. The simulation snapshots for (**a**) NH_3_, (**b**) -NH_2_, and (**c**) -NH-, respectively.

The results for DV(555-777) are given in Fig. 3. One can see that both the -NH_2_ and -NH- can be well trapped by the defect, but the NH_3_ can not, suggesting that the occurrence of the first step of NH_3_ decomposition in the thermal treatment experiments does not need the help of the defect. Our simulations also show that although the -NH_2_ and -NH- can be trapped by the defect, both of them can not dehydrogenate at all, even the simulation time has been extended to 3 ps. It is well known that without dehydrogenation, the formation of N-doping is impossible.

**Figure 4:**
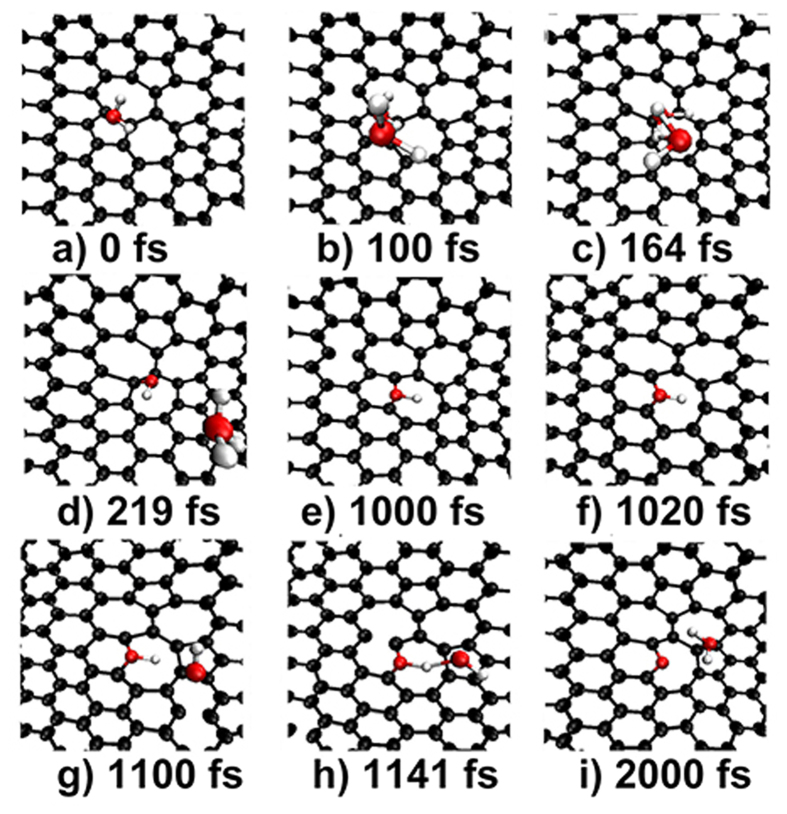
Generating an atomic-N at the DV(555-777). (**a–e**) Simulation snapshots of the dehydrogenation process for -NH_2_ to -NH- and (**f–i**) for -NH to atomic-N via the interaction of multiply N-containing groups.

We extended our simulations to consider the possible dehydrogenation from the interaction of N-containing groups, and the results are given in Fig. 4. Interestingly, more than two N-containing groups can be successively trapped by a DV(555-777) and the dehydrogenation occurs due to the reaction of the multiply trapped N-containing groups. One can see that once the second -NH_2_ appears at 100 fs, it moves to the defect region and reacts with the first one to form a -NH- and a NH_3_. The formed NH_3_ drifts freely above the surface and runs out of the sight as expected. While the formed -NH- adsorbs tightly at the bridge-site of the first 5–7 pair as shown in Fig. 4(e). Subsequently, the -NH- reacts with another approaching -NH- to form an atomic-N and a -NH_2_. The formed atomic-N transforms to a pyridinic-N due to merged into the 5–7 pair. Instead of drifting away, he -NH_2_ moves quickly to the second second 5–7 pair to adsorb on a top-site.

Remarkably, a DV(555-777) can trap multiply N-containing groups which induce a two-step dehydrogenation process that leads to the formation of an atomic-N. It can be simply represented by the “-NH_2_ → -NH- → atomic-N” route.

**Figure 5:**
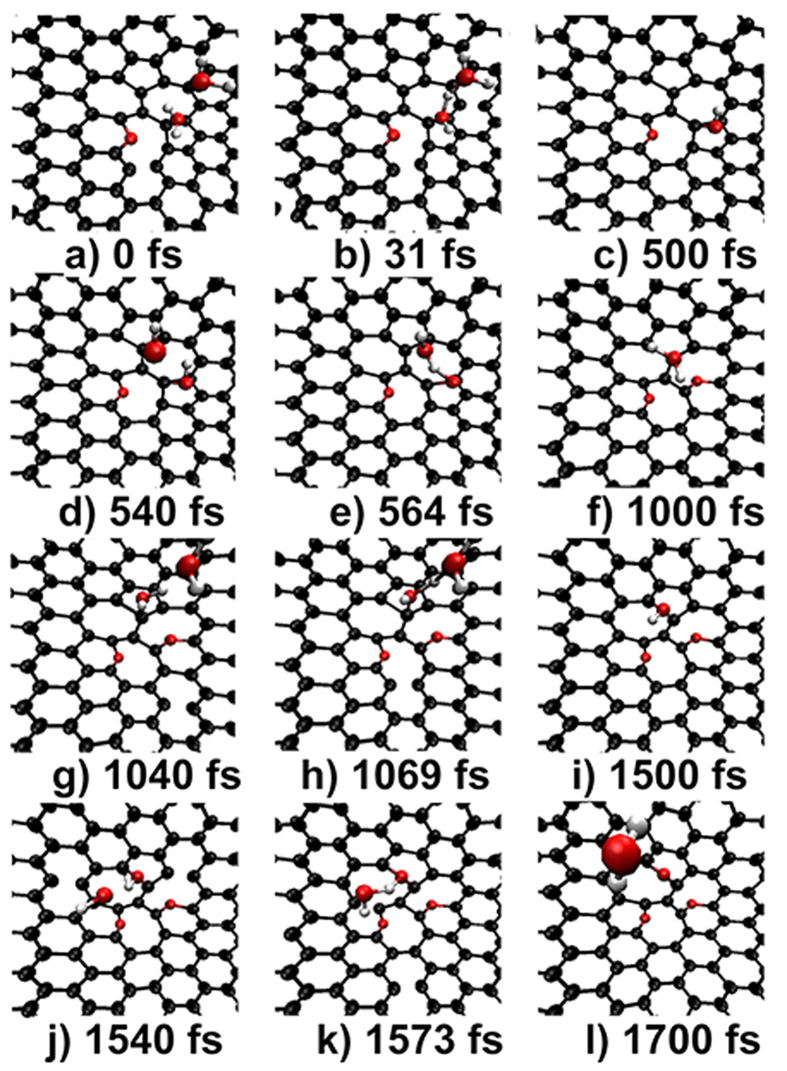
Generating the other two atomic-Ns. Simulation snapshots for the dehydrogenation process to form the second atomic-N (**a–f**) and the third atomic-N (**g–l**).

We found that the process continues as more N-containing groups appear at the reaction region, making as many as three atomic-Ns to be formed at the vicinity of a DV(555-777). One can see in Fig. 5 that the second atomic-N is formed at the second 5–7 pair via another round of dehydrogenation as demonstrated in Fig. 5(a–f), the third atomic-N is formed at the third 5–7 pair via the third round of dehydrogenation as shown in Fig. 5(g–l), and the dehydrogenation process stops due to that the formed -NH_2_ at the third round moves away from the surface of graphene. Surprisingly, although to heal a DV(555-777) requires only two adatoms, as many as three atomic-Ns can be formed at the vicinity.

**Figure 6:**
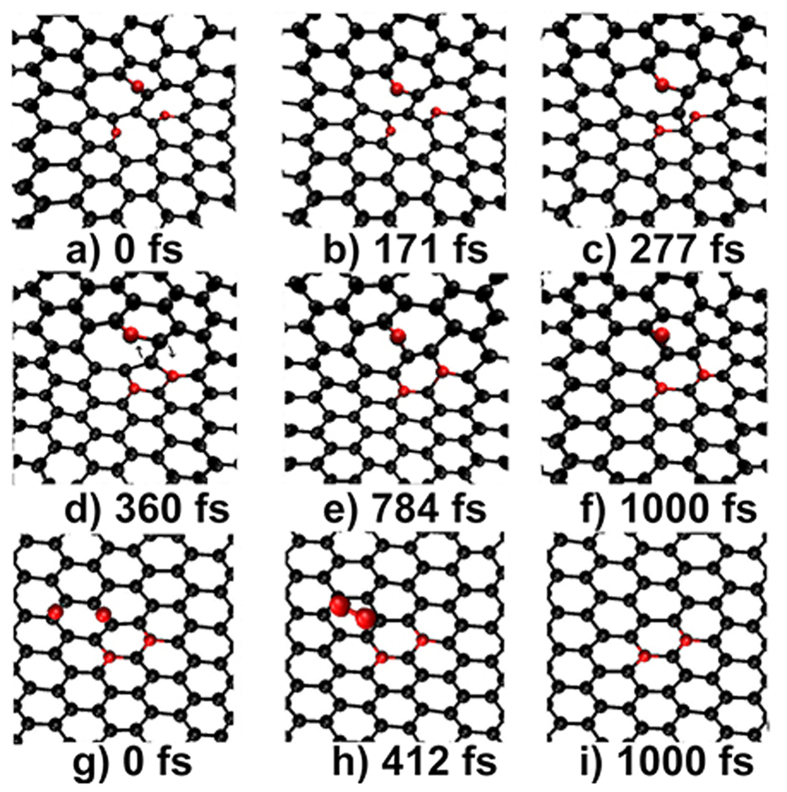
Formation of 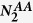 via a complex atomic rearrangement process at the DV(555-777). Simulation snapshots for the atomic rearrangement process (**a–f**) to produce 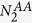 from the two pyridinic-Ns, and the desorbing process (**g–i**) of the third atomic-N which participates the process as a catalyst. The third atomic-N has the same species (bridge-N) at the initial stage (**a**) and final stage (**f**) of the rearrangement.

As the simulation proceeds further, we found the significance of the coexistence of three atomic-Ns at a divacancy. It is that a complex atomic rearrangement process can be induced, directly resulting in the formation of graphitic-Ns and the healing of the defect. The entire process involves the rotation, break, and formation of multiply bonds as shown in Fig. 6(a–f). At the initial stage, the three atomic-Ns present in two pyridinic-Ns and one bridge-N. The latter incorporates into graphene layer to form a pyridinic-N at 171 fs that launches the rearrangement process. One can see that a 5-member ring is formed at 360 fs due to the formation of two C-N bonds and the break of a C-C bond. Then, the 5-member ring transforms to a 6-member ring due to the rotation of a C-C bond of 90° as denoted in Fig. 6(d). The two pyridinic-Ns first merge into the 5-member ring and then become two graphitic-Ns due to that the 5-member ring has changed to the 6-member ring. The two graphitic-Ns quasi-adjacently occupy the same sublattice (A) of graphene with a doping configuration of 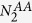 12. While the third N is squeezed out the graphene network and transforms back to a bridge-N as shown in Fig. 6(f).

It is worthy of noting that the third N has the same species (bridge-N) at the initial and final stages of the rearrangement. While Figure S2 have shown clearly that without the third N, atomic rearrangement cannot be launched and the two trapped N atoms cannot transform to graphitic-Ns at all. This means that the bridge-N acts as a catalyst to reduce the energy barrier of the rearrangement. To check this, we have performed simulations to model the interaction of a DV(555-777) direct with three N atoms. One can see in Figure S3 that the three N atoms are trapped by the DV(555-777) and an atomic rearrangement process also happens. Although the initial model and the intermediate process are different from the case of N-containing groups, the conclusion is the same, namely two graphitic-Ns with configuration 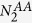 are formed and a bridge-N is participated in the rearrangement process as a catalyst.

As a catalyst, the bridge-N should be active and unstable at experimental conditions. This is why bridge-N has never been observed in the N-graphene obtained in experiments. We have performed density functional theory (DFT) calculations and found that the energy barrier for process of “bridge-N → top-N → bridge-N” is about 1.15 eV when considered vdW corrections. This means that the bridge-N migration at the surface of graphene can occur easily at macroscopic time range of the real thermal treatment experiments, but the probability to find the migration in fs-ps time range simulations should be very small. Thus we have only considered what will happen when two bridge-Ns have moved close to each other. The results displayed in Fig. 6(g–i) show that the bridge-N desorbs by the formation of a nitrogen molecule. Eventually, only the two graphitic-Ns with configuration 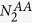 survive in the N-graphene and the defect is healed simultaneously.

**Figure 7:**
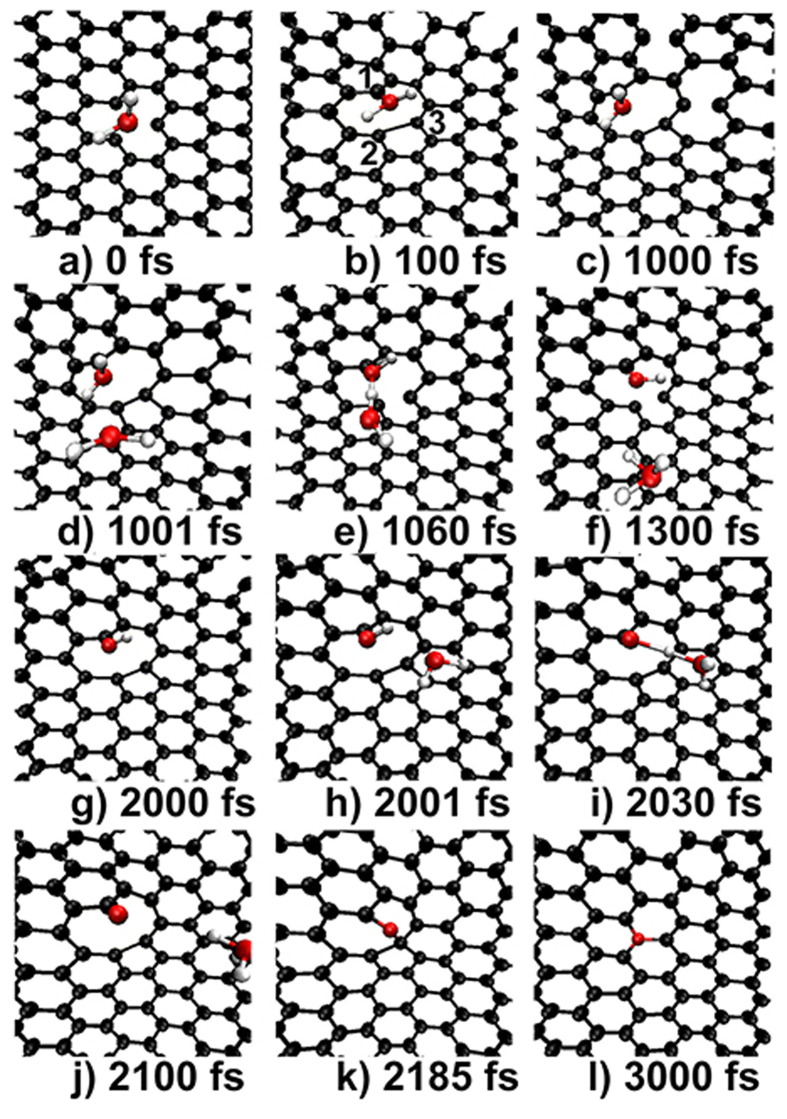
Formation of *N*_1_ at the SV(5–7). (**a–c**) trapping a -NH_2_ at the top-site, (**d–g**) dehydrogenating the -NH_2_ to a -NH, (**h–j**) dehydrogenating the -NH to an atomic-N, and (**k,l**) generating of *N*_1_ from the atomic-N.

We have further extended our simulations to consider the possible formation of graphitic-N at the defect of SV(5-9) and the snapshots are given in Fig. 7. One can see that multiply N-containing groups can also be trapped by a SV(5-9), the dehydrogenation process occurs following the same route of “-NH_2_ → -NH- → atomic-N”, and the result is also basically the same, namely a single graphitic-N (with a configuration of *N*_1_) is formed eventually that heals the defect perfectly.

## Discussion

### Importance of the localized force field

Our simulations show that among the native point defects of graphene, only the SV(5-9) and DV(555-777) have the ability to trap N-containing groups and to mediate the subsequent N-doping process. This can be well understood by analyzing the localized force field of the defects.

Unambiguously, there exist two major localized force fields at a defect: a strain field (S-field) caused by lattice-disorder and an electronic field (E-field) generated due to the existence of dangling bonds. From a structural point of view, a SW(55-77) is formed by the rotation of a C-C bond of 90° at the perfect region of graphene, thus only the S-field presents at the vicinity. Our simulations show that the S-field of SW(55-77) is not large enough to trap N-containing groups at experimental conditions. But for SV(5-9), it is formed by missing a C atom. There exist three dangling bonds at the vicinity. It undergoes a Jahn-Teller distortion which can lead to the saturation of two of the three dangling bonds, but one dangling bond always remains owing to geometrical reasons. Thus both the S-field and the E-field exist at a SV(5-9), resulting in the localized force field which is large enough to trap N-containing groups.

While for DV(5-8-5) and DV(555-777), they both form by missing a pair of C atoms. Self-saturations let no unsaturated C atoms but only lattice-disorder present at the defect. It is well known that a DV(555-777) can be formed from a DV(5-8-5) by the rotation of a C-C bond of 90° 26,35. The rotation decreases the nanohole size but increases the lattice-disorder region largely, resulting in that the force field of DV(555-777) is larger than that of DV(5-8-5). Cretu *et al.*^34^ have found from first principles calculations that the force field of DV(555-777) can generate a large energy gradient (EG) extending widely at the vicinity. Our simulations demonstrate that the large EG plays a critical role in trapping N-containing groups and mediating the subsequent N-doping process.

We noticed that the trapped N atom adsorbs on the top-site of SV(5-9) (see Fig. 2(c)), but on the bridge-site of DV(555-777) (see Fig. 2(e)). The different adsorption sites evidence that the different types of force field of the two defects to direct and trap N-sources.

### Importance of N-containing groups interaction

Our simulations show that the -NH_2_ and -NH- can not dehydrogenate when only considered the interaction of the N-containing group with graphene, in consistent with the observations of previous studies^8^,^27^,^32^. While, as the possibility to trap multiply N-containing groups by a defect is considered, a two-step dehydrogenation route of “-NH_2_ → -NH- → atomic-N” is revealed in our simulations. It is clear that multiply N-containing groups can be trapped by a SV(5-9) or a DV(555-777), and the interaction between them demonstrates the same route of dehydrogenation at the two defects. This means that the revealed dehydrogenation route is independent on the type of the defects but dominated by the interaction of N-containing groups.

### Importance of the structural symmetry of DV(555-777)

Our simulations show that without the third atomic-N, the two atomic-Ns can not launch the complex atomic rearrangement process and the N-doping of 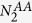 can not be formed at the DV(555-777), suggesting the importance of the third atomic-N for the formation of N-doping. It is understandable that two atomic-Ns can be formed at a DV(555-777), since the DV is formed by missing two C atom. But why as many as three atomic-Ns can be formed at the vicinity of a DV(555-777) needs to be further addressed.

It is well known that a DV(555-777) contains four disordered C atoms of, one locates at the center and the other three surround it, resulting in the formation of three 5–7 pairs in *D*_3_*h* symmetry. First principles calculations have confirmed that the localized force field of a DV(555-777) extends widely at the vicinity in *D*_3_*h* symmetry^34^. Our simulation of modeling the direct interaction of a DV(555-777) with three N atoms (see Figure S3) have shown that the three N atoms simultaneously move towards to the defect region and adsorb on the three 5–7 pairs respectively to form three pyridinic-Ns. It is clear that the specific *D*_3_*h* symmetry allows three atomic-Ns to be formed at the vicinity of a DV(555-777). The symmetry plays a crucial role in determining the number of the formed atomic-Ns and mediating the formation of 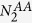 at the DV(555-777).

### The defect-configuration relationship

Experimental measurements show that the formed graphitic-Ns can either distribute randomly between the two sublattices of graphene^7^,^13^ or exhibit well-segregated domains in the same sublattice^13^. A single graphitic-N has doping configuration of N_1_ and two graphitic-Ns quasi-adjacently occupied the same sublattice (A) of graphene result in the doping configuration of 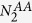 12,29. Different configurations correspond to different electronic structures and applications. Especially, Lv *et al.*^12^ have reported that most of N-dopants (80%) in their N-graphene obtained via thermal treatment of CVD-prepared graphene with NH_3_ are 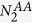 that has much enhanced molecular sensing ability. However, how such an important doping configuration is formed was totally unknown.

Our ab-MD simulations have certainly resolved this long-lasting issue. Basis on our simulations, the relationship between the native point defects of graphene and the doping configurations of the graphitic-N can be established. Namely, SV(5-9) and DV(555-777) mediate to the formation of N_1_ and 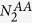, respectively, and the other native point defects can not mediates to the formation of graphitic-N directly.

The finding from our ab-MD simulations is of importance. It reveals for the first time that the DV(555-777) is a favorable active site for N-doping. This is also a somewhat expected result when considering the observation from previous studies^26^ that a DV(555-777) has a lower formation energy than a SV(5-9) or a DV(5-8-5), and a DV(555-777) can be formed by the diffusion and coalescence of two SV(5-9) or by the transformation of a DV(5-8-5) with the rotation of a C-C bond of 90°. First principle calculations have shown that the energy barrier for a SV(5-9) migration in graphene is about 1.3 eV^36^ and for the transformation of DV(5-8-5) to DV(555-777) is about 5.17 eV^35^. Thus, although both the coalescence and transformation processes can not been observed in our fs-ps time range of simulations, a large amounts of DV(555-777) can be expected to exist in the pre-prepared graphene, resulting in the formation of many 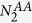. First-principles calculations^12^ show that the formation energy of a 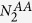 is actually higher than that of a N_1_ or a 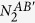. Our simulations illustrate that the thermodynamics and kinetics are essential in understanding the formation mechanism of N-graphene with a higher formation energy. Based on our observations and the fact that SVs and DVs are the main point defects in irradiated graphene^26^,^37^, we propose to improve the content of graphitic-N in N-graphene through introducing extra vacancies by spatially selective irradiation before performing N-doping.

In conclusion, by performing *ab initio* molecular dynamic simulations, we have found a new mechanism for substitutional nitrogen-doping in thermally treated graphene that satisfactorily explains the experimental observations and will be helpful for realizing targeted synthesis of nitrogen-graphene with reduced defects and desired doping configurations.

## Methods

A large supercell (15 × 15 × 1) containing 450 C atoms (see Figure S1) was adopted in simulations to eliminate the periodic interactions. Considered that ammonia is a widely used N-precursor for N-doping and the decomposition of ammonia at the experimental temperature (typically in the range from 700 to 1000 °C) is complicated, NH_3_ molecule and its decomposing products of -NH_2_, -NH-, and -N were used as the possible N-containing groups for N-doping. The initial position of the containing group was set to 3.6 Å away from the graphene layer to ensure the elimination of direct chemical interactions. In this case, vdW forces play the dominant role at the initial stage of simulations.

The simulations were carried out using ab initio package SIESTA 3.2^38^. A Nose-Parrinello-Rahman thermostat was employed with the constant temperature of 850 °C for most of simulations. Electronic structures were calculated using density functional theory (DFT) including vdW corrections (vdW-DFT). The exchange-correlation potential was described with the revised Perdew-Burke-Ernzerhof (rPBE) functional of the generalized-gradient approximation (GGA)^39^. The double *ζ* polarization (DZP) numerical atomic orbital basis sets with the energy cut-off of 80 meV were adopted. The real-space integration was done on the mesh defined by 200 Ry kinetic energy cutoff. Each step was achieved with the tolerance of density matrix (DM) of 1.0^−4^. The snapshots of the simulations were grabbed using VMD program^40^ with the display window close to the active site for clarity.

## Additional Information

**How to cite this article**: Li, X.-F. *et al.* Unraveling the formation mechanism of graphitic nitrogen-doping in thermally treated graphene with ammonia. *Sci. Rep.* **6**, 23495; doi: 10.1038/srep23495 (2016).

## Supplementary Material

Supplementary Information

Supplementary Information

Supplementary Information

Supplementary Information

Supplementary Information

Supplementary Information

Supplementary Information

## Figures and Tables

**Figure 1:**
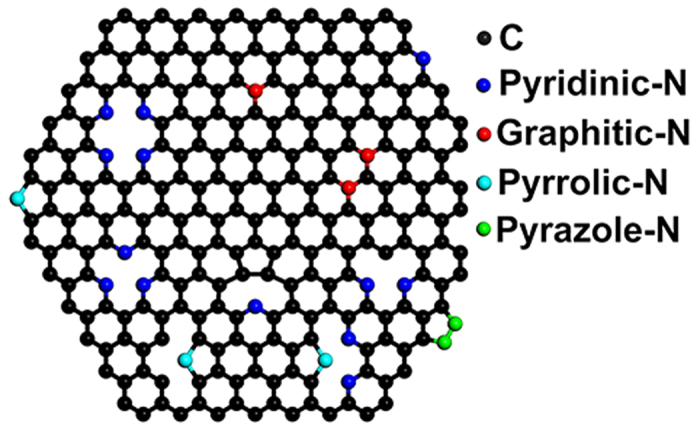
Schematic drawings of the different N-species formed in graphene. Graphitic-Ns are formed in the graphene network, here only the single substitution (N_1_) and double substitution (with configuration 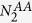) are given. While, other N-species are formed at the edge of nanoholes or the edge of graphene. For simplicity, the hydrogen atoms for saturating dangling bonds are not shown.

## References

[b1] Novoselov K. S. *et al.* A roadmap for graphene. Nature 490, 192–200 (2012). 23060189 10.1038/nature11458

[b2] Wang T., Wang L.-X., Wu D.-L., Xia W. & Jia D.-Z. Interaction between nitrogen and sulfur in co-doped graphene and synergetic effect in supercapacitor. Sci. Rep. 5, 9591 (2015). 25880811 10.1038/srep09591 PMC5381751

[b3] Lin T. *et al.* Nitrogen-doped mesoporous carbon of extraordinary capacitance for electrochemical energy storage. Science 350, 1508–1513 (2015). 26680194 10.1126/science.aab3798

[b4] Tang P. *et al.* The microwave adsorption behavior and microwave-assisted heteroatoms doping of graphene-based nano-carbon materials. Sci. Rep. 4, 5901 (2014). 25109492 10.1038/srep05901 PMC4127501

[b5] Zhao L. *et al.* Visualizing individual nitrogen dopants in monolayer graphene. Science 333, 999–1003 (2011). 21852495 10.1126/science.1208759

[b6] Zhang Y. *et al.* Manageable N-doped graphene for high performance oxygen reduction reaction. Sci. Rep. 3, 2771 (2013). 24067782 10.1038/srep02771 PMC3783894

[b7] Wang X. *et al.* N-doping of graphene through electrothermal reactions with ammonia. Science 324, 768–771 (2009). 19423822 10.1126/science.1170335

[b8] Wang H., Maiyalagan T. & Wang X. Review on recent progress in nitrogen-doped graphene: synthesis, characterization, and its potential applications. ACS Catal. 2, 781–794 (2012).

[b9] Wen Z. *et al.* Crumpled nitrogen-doped graphene nanosheets with ultrahigh pore volume for high-performance supercapacitor. Adv. Mater. 24, 5610–5616 (2012). 22890786 10.1002/adma.201201920

[b10] He W., Jiang C., Wang J. & Lu L. High-rate oxygen electroreduction over graphitic-N species exposed on 3D hierarchically porous nitrogen-doped carbons. Angew. Chem. Int. Ed. 53, 9503–9507 (2014). 10.1002/anie.201404333 25044805

[b11] Wang R., Xu C., Sun J. & Gao L. Three-dimensional Fe_2_O_3_ nanocubes/nitrogen-doped graphene aerogels: Nucleation mechanism and lithium storage properties. Sci. Rep. 4, 7171 (2014). 25421070 10.1038/srep07171 PMC4243062

[b12] Lv R. *et al.* Nitrogen-doped graphene: beyond single substitution and enhanced molecular sensing. Sci. Rep. 2, 586 (2012). 22905317 10.1038/srep00586 PMC3421434

[b13] Zabet-Khosousi A. *et al.* Segregation of sublattice domains in nitrogen-doped graphene. J. Am. Chem. Soc. 136, 1391–1397 (2014). 24392951 10.1021/ja408463g

[b14] Cai J. *et al.* Graphene nanoribbon heterojunctions. Nat. Nanotech. 9, 896–900 (2014). 10.1038/nnano.2014.184 25194948

[b15] Li X.-F., Lian K.-Y., Qiu Q. & Luo Y. Half-filled energy bands induced negative differential resistance in nitrogen-doped graphene. Nanoscale 7, 4156–4162 (2015). 25665635 10.1039/c4nr07472f

[b16] Imran Jafri R., Rajalakshmi N. & Ramaprabhu S. Nitrogen doped graphene nanoplatelets as catalyst support for oxygen reduction reaction in proton exchange membrane fuel cell. J. Mater. Chem. 20, 7114–7117 (2010).

[b17] Li J. *et al.* Direct transformation from graphitic C3N4 to nitrogen-doped graphene: An efficient metal-free electrocatalyst for oxygen reduction reaction. ACS Appl. Mater. Interfaces 7, 19626–19634 (2015). 26305578 10.1021/acsami.5b03845

[b18] Guo D. *et al.* Active sites of nitrogen-doped carbon materials for oxygen reduction reaction clarified using model catalysts. Science 351, 361–365 (2016). 26798009 10.1126/science.aad0832

[b19] Bronner C. *et al.* Aligning the band gap of graphene nanoribbons by monomer doping. Angew. Chem. Int. Ed. 52, 4422–4425 (2013). 10.1002/anie.201209735 23512734

[b20] Dong H., Zhao Y., Tang Y., Burkert S. C. & Star A. Oxidative unzipping of stacked nitrogen-doped carbon nanotube cups. ACS Appl. Mater. Interfaces 7, 10734–10741 (2015). 25946723 10.1021/acsami.5b00447 PMC6563925

[b21] Jeong H. M. *et al.* Nitrogen-doped graphene for high-performance ultracapacitors and the importance of nitrogen-doped sites at basal planes. Nano Lett. 11, 2472–2477 (2011). 21595452 10.1021/nl2009058

[b22] Park S. *et al.* Chemical structures of hydrazine-treated graphene oxide and generation of aromatic nitrogen doping. Nat. Commun. 3, 638 (2012). 22273676 10.1038/ncomms1643

[b23] Huang H. *et al.* NH_3_ assisted photoreduction and N-doping of graphene oxide for high performance electrode materials in supercapacitors. Nanoscale 7, 2060–2068 (2015). 25553955 10.1039/c4nr05776g

[b24] Luo Z. *et al.* Pyridinic n doped graphene: synthesis, electronic structure, and electrocatalytic property. J. Mater. Chem. 21, 8038–8044 (2011).

[b25] Zhang S., Tsuzuki S., Ueno K., Dokko K. & Watanabe M. Upper limit of nitrogen content in carbon materials. Angew. Chem. Int. Ed. 54, 1302–1306 (2015). 10.1002/anie.201410234 25424704

[b26] Banhart F., Kotakoski J. & Krasheninnikov A. V. Structural defects in graphene. ACS Nano 5, 26–41 (2011). 21090760 10.1021/nn102598m

[b27] Wang B., Tsetseris L. & Pantelides S. T. Introduction of nitrogen with controllable configuration into graphene via vacancies and edges. J. Mater. Chem. A 1, 14927–14934 (2013).

[b28] Sharifi T., Hu G., Jia X. & Wagberg T. Formation of active sites for oxygen reduction reactions by transformation of nitrogen functionalities in nitrogen-doped carbon nanotubes. ACS Nano 6, 8904–8912 (2012). 23020173 10.1021/nn302906r

[b29] Deng D. *et al.* Toward n-doped graphene via solvothermal synthesis. Chem. Mater. 23, 1188–1193 (2011).

[b30] Kim H., Lee K., Woo S. I. & Jung Y. On the mechanism of enhanced oxygen reduction reaction in nitrogen-doped graphene nanoribbons. Phys. Chem. Chem. Phys. 13, 17505–17510 (2011). 21946759 10.1039/c1cp21665a

[b31] Zhao Y., Nakamura R., Kamiya K., Nakanishi S. & Hashimoto K. Nitrogen-doped carbon nanomaterials as non-metal electrocatalysts for water oxidation. Nat. Commun. 4, 2390 (2013). 23979080 10.1038/ncomms3390

[b32] Wang B. & Pantelides S. T. Controllable healing of defects and nitrogen doping of graphene by CO and NO molecules. Phys. Rev. B 83, 245403 (2011).

[b33] Hou Z. & Terakura K. Effect of nitrogen doping on the migration of the carbon adatom and monovacancy in graphene. J. Phys. Chem. C 119, 4922–4933 (2015).

[b34] Cretu O. *et al.* Migration and localization of metal atoms on strained graphene. Phys. Rev. Lett. 105, 196102 (2010). 21231186 10.1103/PhysRevLett.105.196102

[b35] Lee G.-D. *et al.* Diffusion, coalescence, and reconstruction of vacancy defects in graphene layers. Phys. Rev. Lett. 95, 205501 (2005). 16384068 10.1103/PhysRevLett.95.205501

[b36] Krasheninnikov A. V., Lehtinen P. O., Foster A. S. & Nieminen R. M. Bending the rules: contrasting vacancy energetics and migration in graphite and carbon nanotubes. Chem. Phys. Lett. 418, 132–136 (2006).

[b37] Guo B. *et al.* Controllable N-doping of graphene. Nano Lett. 10, 4975–4980 (2010). 20968305 10.1021/nl103079j

[b38] Soler J. M. *et al.* The siesta method for ab initio order-n materials simulation. J. Phys.: Condens. Matter 14, 2745–2779 (2002).

[b39] Hammer B., Hansen L. B. & Norskov J. K. Improved adsorption energetics within density-functional theory using revised perdew-burke-ernzerhof functionals. Phys. Rev. B 59, 7413–7421 (1999).

[b40] Humphrey W., Dalke A. & Schulten K. VMD–visual molecular dynamics. J. Mol. Graph. 14, 33–38 (1996). 8744570 10.1016/0263-7855(96)00018-5

