# Supplementary material for: Unraveling the formation mechanism of graphitic nitrogen-doping in thermally treated graphene with ammonia
Source: Sci Rep. 2016 Mar 22;6:23495. doi: 10.1038/srep23495 (PMC4802320; doi:10.1038/srep23495)
Supplement: Supplementary Information [file srep23495-s1.pdf]

Supporting Information for

Unraveling the formation mechanism of graphitic nitrogen doping in thermally treated graphene with ammonia

Xiao-Fei Li*[a], Ke-Yan Lian[b], Lingling Liu[a], Yingchao Wu [a], Qi Qiu[a], Jun Jiang[c], Mingsen Deng[d], and Yi Luo*[b,c,d]

[a] *School of Optoelectronic Information, University of Electronic Science and Technology of China, Chengdu, Sichuan, 610054, China*

[b] *Division of Theoretical Chemistry and Biology, School of Biotechnology, Royal Institute of Technology, S-106 91 Stockholm, Sweden*

[c] *Hefei National Laboratory for Physical Sciences at the Microscale and Synergetic Innovation Center of Quantum Information and Quantum Physics, University of Science and Technology of China, Hefei, Anhui 230026, China and*

[d] *Guizhou Synergetic Innovation Center of Scientific Big Data for Advanced Manufacturing Technology, Guizhou Education University, Guiyang, 550018, China*

** e-mail: xf.li@uestc.edu.cn luo@kth.se*

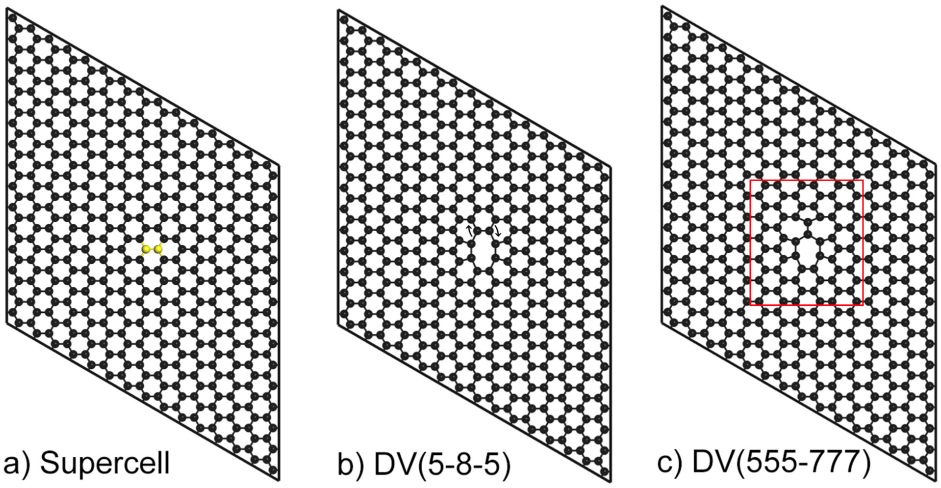

Figure S1 | (a) the large supercell (15×15) contains 450 C atoms. (b) the large supercell contains a divacancy DV(5-8-5) formed by missing a pair of C atoms. (c) the large supercell contains a divacancy DV(555-777) formed from a DV(5-8-5) by rotating a C-C bond of 90 degree. The red rectangle is the display window which has been moved close to the active site for clarity.

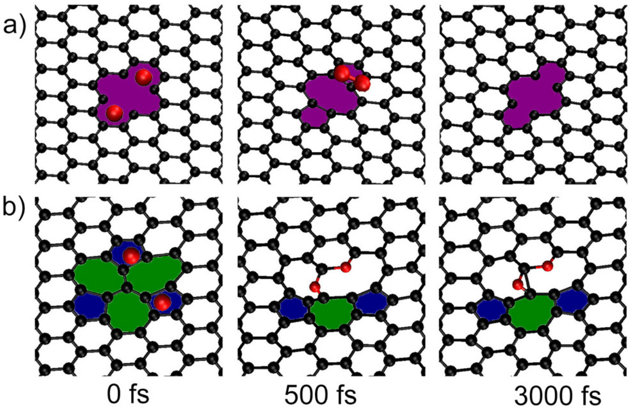

Figure S2 | Simulation snapshots for the interaction of two N atoms with a divacancy. (a) for divacancy DV(5-8-5) and **(b)** for divacancy DV(555-777) . The DV(5-8-5) does not have an ability to trap the two N atoms which form a nitrogen atom and run out of the sight in the simulations. While the DV(555-777) has an ability to trap the two N atoms and to mediate the two N atom to form a pyridinic-N and a bridge-N at the defect region.

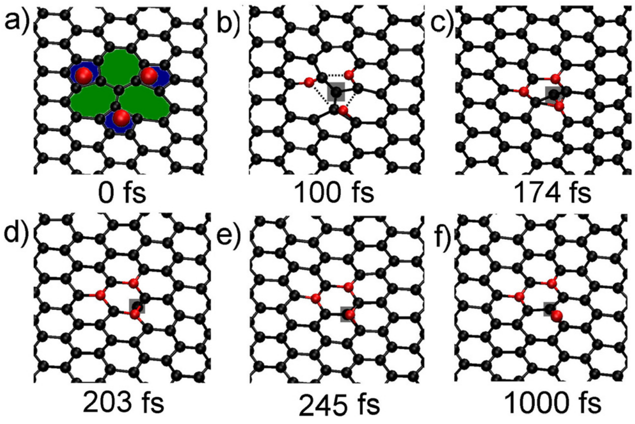

Figure S3 | Simulation snapshots for the interaction of three N atoms with a DV(555-777) . **(a)** the initial structure of the system. **(b)** At 100 fs, two pyridinic-Ns and a bridge-N are formed. **(c)** Then the bridge-N incorporate into graphene layer leading to the transformation of the two pyridinic-Ns to two graphitic-Ns with a configuration of N_2^{AA} . During this process, the central carbon atom of DV(555-777) is pushed out of the graphene layer to the opposite side of the third N to form a dumbbell-like structure. **(d)** After that, the central carbon atom transforms into a bridge-C as one of its three C-C bonds is broken, and simultaneously the third N atom merges into the six-member ring by occupying the same sublattice as the first two N atoms. Temporarily, three graphitic-Ns with doping a configuration of N_3^{AAA} are obtained. **(e)** As the simulation further proceeds, the bridge-C squeezes into the six-member ring, and forces the latest graphitic-N to move out of the graphene layer to become a top-N. **(f)** The remaining two graphitic-Ns with a configuration N_2^{AA} keep the form in the subsequent simulations, while the top-N transforms back to a bridge-N.

S4. The videos for six selected simulation processes.

1_NH2_to_NH: the video of the $-NH_2$ captured by a DV(555-777) and then dehydrogenating to a -NH- via reacting with another approaching $-NH_2$.

2_NH_to_atomic-N: the video of the -NH- captured by a DV(555-777) and then dehydrogenating to an atomic-N via reacting with another approaching -NH-.

3_atomic_Ns_to_graphitc_Ns_at_DV: the video of the dehydrogenation process of generating three atomic-Ns at the vicinity of a DV(555-777) and the atomic rearrangement process for the formation of two graphitic-Ns.

4_NH2_to_single_graphitic_N_at_SV: the video of forming a single graphitic-N at the SV(5-9).

5_ N_atoms_to_ graphitic_N: the video of forming two graphitic-Ns by the reaction of three N atoms directly with a DV(555-777).

6_remove_bridge-N: the video of removing the bridge-N by the formation of a nitrogen molecule.
